# Tailoring acceptance and commitment therapy for parents of children with undiagnosed conditions: a qualitative pre-implementation study

**DOI:** 10.1186/s13023-026-04355-w

**Published:** 2026-05-02

**Authors:** Sarah Rutzick, Sharon Aufox, Elizabeth Borto, Erika Lawrence, Kelli Scott, Rowan Forbes Shepherd, Carly Rasmussen, Jennifer L. Young

**Affiliations:** 1 Center for Genetic Medicine, Feinberg School of Medicine, Northwestern University, Chicago, IL USA; 2 Department of Obstetrics and Gynecology, Feinberg School of Medicine, Northwestern University, Chicago, IL USA; 3 The Family Institute, Northwestern University, Evanston, IL USA; 4 Department of Psychology, Northwestern University, Evanston, IL USA; 5 Center for Dissemination and Implementation Science, Feinberg School of Medicine, Northwestern University, Chicago, IL USA; 6 Department of Medical Social Science, Feinberg School of Medicine, Northwestern University, Chicago, IL USA; 7 Division of Cancer Epidemiology and Genetics, National Cancer Institute, National Institutes of Health, Rockville, MD USA; 8 Division of Genetics, Genomics, and Metabolism, Ann & Robert H. Lurie Children’s Hospital of Chicago, Chicago, IL USA

**Keywords:** Undiagnosed disease, Psychosocial intervention, Acceptance and commitment therapy, Pre-implementation research, Parent support

## Abstract

**Background:**

Parents of undiagnosed children (POUC) experience significant psychosocial challenges, including anxiety, uncertainty, and isolation, that stem from parenting medically complex children while facing obstacles throughout the diagnostic journey. Despite these well-described challenges, a mental health intervention designed to meet the unique needs of POUC, which is necessary to promote the psychological and overall wellbeing of this population, does not exist. Acceptance and Commitment Therapy (ACT) has proven effective in a wide range of populations and shows promise for POUC. With the goal of designing and implementing an ACT-based intervention tailored to POUC, this pre-implementation study aimed to understand their psychosocial needs and prior mental health support experiences, explore their reactions towards ACT, and determine their anticipated barriers, facilitators, and preferences for participating in an ACT skills group, guided by the Consolidated Framework for Implementation Research (CFIR).

**Methods:**

Semi-structured, individual interviews were conducted with 18 POUC, including an experiential portion that exposed participants to key ACT concepts and exercises. Inductive coding based on participant responses and deductive coding based on the CFIR were employed to code interview transcripts. Reflexive thematic analysis was performed to identify key findings.

**Results:**

Isolation was a psychosocial challenge for which all participants desired support. Many participants reported inadequacies in their prior mental health support, primarily due to lack of understanding from therapy providers regarding their unique circumstances. Although most participants indicated that ACT could help them manage difficult thoughts and emotions and act in alignment with their values, they also described achievability, collaboration, and accountability as key elements that could support their uptake. The main barriers, facilitators, and preferences that participants highlighted were related to group design (accessibility, flexibility) as well as their own characteristics as recipients (capability, need, and motivation).

**Conclusions:**

This pre-implementation study affirmed the potential value of ACT for POUC and identified key opportunities for tailoring an ACT skills group to meet their needs. Future research, including pilot implementation studies, are needed to evaluate the effectiveness of a tailored ACT skills group and further refine both the intervention and its implementation strategy.

**Supplementary Information:**

The online version contains supplementary material available at 10.1186/s13023-026-04355-w.

## Introduction

Diagnosis is central to the practice of medicine, but is not guaranteed, especially for rare conditions. Rare conditions, or those impacting fewer than 200,000 individuals, collectively affect approximately 6–8% of the United States population [1]. Undiagnosed conditions are rare conditions that are refractory to standard clinical and laboratory evaluations, including genetic testing, despite the belief that about 80% of undiagnosed conditions have underlying genetic etiologies [1]. Although whole exome sequencing (WES) and whole genome sequencing (WGS) have improved diagnostic yield within recent years, 40–75% of those who undergo WES or WGS remain undiagnosed [1]. These individuals may miss out on clinical and personal benefits such as condition-specific management and support, prognostic foresight, recurrence risk information, and enhanced coping abilities [1, 2]. Since symptoms of undiagnosed conditions often emerge early in life, approximately 50% of those affected are children [3].

Existing research has described the psychosocial impacts of parenting a child with an undiagnosed condition. Many challenges mirror those faced by parents of children with *diagnosed* rare diseases, but the absence of a genetic diagnosis introduces distinct and compounding stressors. Commonly reported emotional responses include distress, anxiety, uncertainty, overwhelm, isolation, anger, frustration, shame, guilt, diminished sense of self, and disconnection from others [3–9]. These parents are often required to coordinate care with many different medical providers for their child, who may have significant developmental, intellectual, and/or physical delays and disabilities [5, 6]. Additionally, parenting responsibilities frequently necessitate adjustments to their personal and professional lives, often at the expense of their individual interests, social engagement, and overall well-being [5, 6]. These parents can also struggle with the inability to engage in typical parenting roles or relate to parents without medically complex or undiagnosed children. The absence of a genetic diagnosis in particular limits access to condition-specific information or support and causes uncertainty surrounding prognosis, risks for additional complications, and implications for other family members [4, 7]. Furthermore, parents are often required to repeatedly recount their child’s medical history, endure inappropriate or delayed treatments, and face skepticism or dismissal from healthcare professionals—experiences that compound emotional anguish and contribute to a sense of invisibility within the healthcare system [9].

Although these experiences are well-documented, there is a lack of structured support available to address the unique psychosocial challenges faced by parents of undiagnosed children (POUC). As such, we propose the use of Acceptance and Commitment Therapy (ACT), an evidence-based psychotherapy approach, to support this population [10, 11]. ACT is rooted in the belief that psychological inflexibility and attempting to suppress unpleasant thoughts or emotions increases psychological distress. Thus, ACT aims to enhance psychological flexibility by promoting present-moment engagement and values-driven living. Key components of ACT include acceptance—developing willingness to experience difficult thoughts and emotions; mindfulness—becoming aware of present-moment thoughts and emotions; cognitive ‘defusion’—creating distance from distressing thoughts; values—identifying what is important; and committed action—pursuing meaningful actions aligned with those values [10, 11]. ACT has a flexible format with components like metaphors, experiential exercises, and behavior change strategies [10, 11]. Although ACT was originally designed to help individuals with anxiety disorders, depression, and substance use disorders, it has since been effectively tailored to serve a variety of populations and contexts, including parents of medically complex children [12, 13]. In fact, a systematic review by Li et al. (2023) assessing ACT-based interventions in parents of children with special healthcare needs found that ACT had positive effects on parents’ mental health, psychological flexibility, mindfulness abilities, confidence, and self-efficacy [13].

Since the psychosocial challenges faced by POUC closely resemble those of parents of children with special healthcare needs, ACT may offer similar benefits for this population. For example, acceptance practices such as mindfulness and cognitive ‘defusion’ may empower POUC to effectively navigate the overwhelming thoughts and emotions that arise during the diagnostic journey, while committed, value-driven actions may enable them to stay engaged in meaningful aspects of their own lives, even as they support their child through the journey. Li et al. (2023) further identified that group-based ACT interventions were more beneficial than individual ones for parents of children with special healthcare needs [13]. Other studies have compared the effectiveness of group counseling to individual counseling for parents, including a study by Danino and Shechtman (2012) that found group counseling to offer distinct advantages over individual coaching for parents of children with learning disabilities. The group setting not only provided emotional support through shared experiences but also fostered skill development and coping strategies [14]. Studies have underscored the impact of peer support for parents of children with special healthcare needs, finding that it can increase emotional and informational support, reduce social isolation, enhance sense of belonging, and improve both short- and long-term well-being [15, 16]. Taken together, these findings suggest that a tailored intervention coupling the value of peer support with the psychotherapeutic advantages of ACT, such as an ACT skills group, may be effective in addressing the psychosocial challenges of POUC.

Before introducing an ACT skills group for POUC, it is essential to conduct research grounded in implementation science. Implementation science is “the scientific study of methods to promote the systematic uptake of research findings and other evidence-based practice into routine practice” [17]. This research will inform the development of a targeted, context-specific implementation strategy that accounts for the multifaceted physical and psychological challenges faced by POUC. One specific implementation framework, the Consolidated Framework for Implementation Research (CFIR), helps researchers identify barriers and facilitators contributing to the implementation of an innovation within a specific context [18]. The CFIR defines five key domains, each containing multiple constructs, that influence implementation: the innovation, or the “thing” being implemented (i.e., ACT); the individuals, or the roles and characteristics of those involved with the innovation (i.e., group facilitators, participants); the inner setting, or the setting in which the innovation is implemented (i.e., the hospital or clinic); the outer setting, or the broader setting in which the inner setting exists (i.e., state or federal policies); and the implementation process, or the activities and strategies used to implement the innovation (i.e., training, supervision) [18]. Guided by the CFIR framework, the aims of this study were to (1) understand the thoughts and emotions for which POUC most desire support, (2) assess their prior experiences receiving mental health support, (3) explore their reactions to ACT concepts and exercises, and (4) determine barriers, facilitators, and their preferences for participating in a tailored ACT skills group. The long-term goal of this research is to develop and deliver a targeted psychotherapy intervention that addresses critical support gaps for this population, ensuring it is contextually appropriate, acceptable, and accessible.

## Methods

### Study design

This pre-implementation study employed qualitative interviewing to assess the perspectives of POUC towards an ACT skills group tailored to meet their unique psychosocial needs. The Consolidated Criteria for Reporting Qualitative Research (COREQ) was consulted to inform research reporting [19]. We approached this study from a subtle realist, interpretivist epistemological stance, recognizing that participants’ experiences reflect a real phenomenon but are accessed through subjective accounts and co-constructed through reflexive interpretation of the research team [20]. The research team was comprised of individuals with relevant expertise in qualitative methods, psychosocial genetics research, implementation science, psychotherapy, ACT intervention development, and genetic counseling for those impacted by undiagnosed conditions.

### Participants and recruitment

Individuals eligible to participate in this study were parents to a child < 18 years old with non-diagnostic WES or WGS results who identified with having an undiagnosed child. They were also required to speak English fluently, have access to Zoom videoconferencing software, and reside in the United States. A recruitment flyer explaining the purpose of the research and the eligibility criteria was distributed across three recruitment sources: (1) The Reassess, Analyze, Research, Empower program at Ann & Robert H. Lurie Children’s Hospital of Chicago, a clinical research program for children with a suspected genetic diagnosis despite exhaustive non-diagnostic testing, (2) The Rare and Undiagnosed Network, an advocacy organization dedicated to those impacted by undiagnosed conditions, and (3) The Undiagnosed Disease Network Foundation Participant Engagement and Empowerment Resource, a resource for individuals accepted into the Undiagnosed Disease Network as well as their family members.

The recruitment flyer contained a link and QR code directing potential participants to a Qualtrics screening survey where, if eligible, they electronically signed the consent form and provided their demographic and contact information. Those selected to participate were called by phone within two days of their survey responses and asked to briefly describe their child’s genetic testing process to confirm eligibility and provide availability for their interview. Participants received a $50 gift card upon completion of their interview.

### Data collection

Individual interviews were conducted by SR, a genetic counseling graduate student with counseling training and experience working in psychotherapeutic settings, between October 2024 and January 2025. Interviews took place over Zoom videoconferencing software, which enabled audio and video recording. Only the audio was transcribed and analyzed. A semi-structured interview guide was developed by the research team based on the study aims. The interview guide was divided into three sections and designed to last approximately 45 min. First, participants were asked to describe the thoughts and emotions for which they would most desire support and, if applicable, recount their prior experiences receiving mental health support, including psychotherapy. Second, participants were led through a short introduction to key ACT concepts and exercises to explore their initial reactions. Material for this portion of the interview guide was adapted from ACT intervention protocols that are published and accessible for a fee on the Association for Contextual Behavioral Science (ACBS) website [21]. This section was split into two parts: (1) acceptance and cognitive ‘defusion,’ and (2) values and committed action. These concepts and exercises were chosen because the research team deemed that they were central to understanding ACT and could be delivered in an abbreviated format given the limited timeframe of the interview. Third, participants were asked to anticipate barriers, facilitators, and their preferences for participating in an ACT skills group, including their preferred setting, format, length, frequency, timing, and referral mode.

### Data analysis

Interviews were transcribed verbatim and uploaded to Dedoose Version 9.2.14 [22] to facilitate qualitative content analysis. A preliminary codebook was drafted utilizing a team-based, reflexive qualitative analysis approach [23]. Three research team members (SR, EB, and JLY) independently open coded two transcripts. These coders met to discuss and develop codes, practicing reflexivity and reconciling inconsistencies in data interpretation between coders. Inductive codes based on participant responses were added to the preliminary codebook. Deductive codes were added based on the study aims as well as the CFIR. For example, a parent code was ‘innovation characteristics,’ corresponding to the innovation domain of CFIR, and a child code under ‘innovation characteristics’ was ‘accessibility,’ a common idea that arose in participant responses. The entire research team reviewed the preliminary codebook and their feedback was incorporated.

Once the preliminary codebook was completed, two coders (SR and EB) independently applied the codebook to two additional transcripts and utilized an iterative memo-ing process throughout. The coders met to compare codes and memos, refining codes until a consensus codebook was finalized. Inter-rater reliability was established between the coders using Dedoose code application tests. The average pooled Cohen’s Kappa was 0.89, indicating very good agreement [24]. One coder (SR) applied the final codebook to the remaining transcripts. The second coder (EB) independently coded every third transcript, comparing their coding with the first coder to ensure continued reliability. The research team utilized a codebook thematic analysis approach: reviewing code applications, translating codes into themes, and coming to consensus via discussion about the final themes [25].

## Results

**Table 1:** Participant characteristics (*n* = 18)

Characteristics	*n* (%)
Age (years)	
Median	43
Range	26-55
Total number of children	
1	2 (11)
2	7 (39)
3	7 (39)
4	2 (11)
Parent	
Mother	17 (94)
Other	1 (6)
Gender	
Woman	17 (94)
Other	1 (6)
Marital status	
Married	15 (83)
Separated	2 (11)
Divorced	1 (6)
Race^a^	
White	16 (89)
Black or African American	1 (6)
Asian	1 (6)
American Indian or Alaska Native	1 (6)
Highest degree completed	
High school	4 (22)
Some college	2 (11)
Associate	2 (11)
Bachelor’s	5 (28)
Master’s	1 (6)
Professional	2 (11)
Doctoral	2 (11)
Employment status	
Working	12 (67)
Not working	5 (28)
Prefer not to say	1 (6)
Geographic location in US	
Midwest	12 (67)
Northeast	1 (6)
West	1 (6)
Southwest	2 (11)
Southeast	2 (11)
Recruitment source	
LCH	6 (33)
RUN	1 (6)
UDNF PEER	11 (61)

^a^Participants were able to select more than one race category. One participant selected two race categories

Abbreviations: LCH = Ann & Robert H. Lurie Children's Hospital of Chicago; RUN = Rare and Undiagnosed Network; UDNF PEER = Undiagnosed Disease Network Foundation Participant Engagement and Empowerment Resource

**Table 2:** Undiagnosed child characteristics (*n* = 18)

Characteristics	*n* (%)
Age (years)	
Median Range	9.5 1–17
Time since most comprehensive GT	
Less than 1 year 1–3 years 3–5 years 5–10 years 10 + years	1 (6) 6 (33) 2 (11) 6 (33) 3 (17)
Most comprehensive GT completed	
WES WGS	9 (50) 9 (50)

Abbreviations: GT = genetic testing; WES = whole exome sequencing; WGS = whole genome sequencing

### Sample

20 individuals completed the screening survey and were eligible to participate. Two participants were lost to follow up; thus, a total of 18 participants were interviewed. Interviews lasted between 31 and 71 min (median 48 min). Table 1 provides a summary of key participant characteristics and Table 2 outlines characteristics of participants’ undiagnosed child and their diagnostic experience. Most participants were recruited from UDNF PEER (66%). Most self-identified as mothers (94%), women (94%), married (83%), white (89%), and working (67%). The highest education attained by participants ranged from high school (22%) to doctoral (11%). While participants interviewed from across the United States, a third of participants resided in the Midwest. Two participants noted that they parented multiple affected, undiagnosed children. In such cases, the information in Table 2 reflects that of their oldest affected child. There were an equal number of participants who had thus far pursued WES and WGS for their undiagnosed child. The amount of time since these tests were completed ranged from < 1 year to > 10 years ago (median 3–5 years ago).

### Overview of major findings

We generated three main findings from participant responses: (1) although participants desired support for a range of psychosocial challenges, they consistently emphasized feelings of isolation and the sense that their unique experiences were not adequately understood or addressed by mental health practitioners; (2) most participants expressed interest in learning more about ACT while also pinpointing targets for tailoring an ACT protocol to best meet their needs; and (3) participants identified the most significant barriers and facilitators to participating in an ACT skills group, which related to both the group’s design and their own individual capability, needs, and motivation to engage—factors that shaped their preferences for implementation.

### Psychosocial challenges

#### POUC struggle with isolation

Participants described an array of psychosocial challenges related to parenting an undiagnosed child for which they desire support. However, all participants expressed feeling isolated in some capacity, and many stated a need for more structured support to address this issue. Participants described three major contributors to isolation in the context of parenting an undiagnosed child: (1) lack of understanding, or the absence of empathy, compassion, validation, or appreciation from people around them, (2) lack of support, or the absence of help, guidance, or resources from medical providers, family members, etc., and (3) lack of community, or the absence of individuals who can relate to or share in their experiences. For example, two participants noted:

*I would probably say the isolation of it all [is the challenge for which mental health support is most desired]. Everyone deals with it in motherhood, but when you have a child who’s undiagnosed, and then the additional opinions on that, it can be so isolating. You don’t feel like anyone really understands, and even in special needs communities, you don’t have a place, like, you don’t fit in, you’re just there. [Participant 9]*

*The average person goes to the doctor, and they have a cold, and they fix it. Or you have a broken bone, and they fix it. And then, all of a sudden, you go to the doctor, and they have no clue what’s going on. They have no answers, and you’re sitting there like, ‘What?’ And you talk to people, and it’s a foreign concept to them, too. [Participant 16]*

#### Understanding from mental health practitioners is key

Interestingly, mental health practitioners often contributed to participants’ feelings of isolation. In fact, lack of understanding from therapists was a key reason participants cited for their prior mental health support experiences falling short. Thirteen participants received prior mental health support in the form of individual therapy, and the perceived level of understanding from the therapist was a determining factor in how they spoke about their therapy experiences. Some participants had worked with therapists who understood the unique difficulties of parenting an undiagnosed child. This quality made these participants feel well supported by their therapists, with two participants indicating that:

*I feel like I have good mental health support, and I’m lucky that my therapist worked as a hospital case manager before she went into private practice. So that gave her a lot of context, I think, for understanding what would happen. [Participant 7]*

*I ended up finding this [therapist] through a community of parents who are raising kids with rare diseases, so I ended up going about it in a very targeted way, and I feel like that has made a big difference in how supported I’m feeling with it. [Participant 10]*

On the other hand, some participants did not feel understood by their therapists, which hindered the therapeutic relationship or caused general dissatisfaction with the therapy experience. For example, two participants reported that:

*I do see a therapist, but I feel like it’s never… I’ve never actually spoken to somebody who wants to honor the fact that this is a unique journey with unique stressors. This is just, like, I see generic therapists, so I feel like it would be more helpful if it was more mindful of the journey that we’ve walked down. [Participant 9]*

*I don’t think I gelled very well with the therapist because she was expecting too much of me. She was like, ‘Oh, I see you as a motivational speaker, and I see you as this, and I see you as that,’ and I was like, ‘Yo, lady, I don’t have time for that.’ And she’s like, ‘Yeah, you really need to get out there and talk to moms and go do this and go do that,’ and I was like, ‘I don’t even have time to brush my teeth or take a shower some days, so no.’ [Participant 14]*

#### POUC seek out understanding among peers

Several participants mentioned engaging with parents in similar situations as a means of informal mental health support, which fostered a sense of community and understanding. One participant articulated the potential benefits of a group for POUC that would allow parents to connect while also promoting healthy coping, noting that:

*…I feel that if there was a chance for a group of people who are experiencing a similar circumstance, that group would be so perfect… I truly think that having these conversations with other people who are experiencing similar circumstances will be very helpful and guide us into not only recognizing what we’re feeling but helping us cope in good ways. [Participant 2]*

In summary, participants reported struggling with various issues related to parenting an undiagnosed child, but isolation was discussed particularly often. Although participants frequently turned to peer support to reduce feelings of isolation, they had less success finding individual psychotherapy that adequately addressed their needs due to perceived lack of understanding from mental health practitioners.

### ACT concepts and exercises

#### POUC were interested in exploring ACT

Most participants immediately recognized the potential value of ACT in the context of parenting an undiagnosed child, and all participants answered affirmatively when asked whether they would be interested in learning more about ACT. After practicing cognitive ‘defusion,’ some participants felt a shift in how they experienced their difficult thoughts. For example, one participant expressed that the cognitive ‘defusion’ exercise helped her move from a sense of guilt to self-compassion:

*It was a huge, an immediate difference in how I felt when I’m thinking, ‘I’m not doing enough,’ between that and, ‘Oh, I have this thought that I’m not doing enough.’ My brain immediately goes to, ‘But I’m doing this, this, and this, and I’m only human. I’m one mom doing all the things that are in my power.’ [Participant 17]*

Additionally, some participants believed that values and committed action could help them prioritize what was most important (their values) and create a feasible, focused plan for moving forward. One participant chose well-being and self-discipline as values she wanted to work on, and articulated that these skills could help create space for her to thrive amongst the uncertainty and daily requirements of parenting an undiagnosed child:

*…as she’s growing, as we move into year nine with no answers, I just have to take the steps and do the things that will allow me to continue to thrive, right? So that I can know joy, too, despite all of this stuff. [Participant 5]*

A few participants struggled to envision how ACT could help. For example, although one participant appreciated the concept of acceptance, they struggled to understand what acceptance means in the context of ACT, particularly for POUC:

*I mean, I love it [the concept of acceptance]. But, I mean, I’m not sure how to accept… I mean, what does it mean to accept, like, and lay back, like, just let it be? Just stop fighting for him? Just stop… like, do whatever I do but not worry about it? I mean, what does it mean to accept? [Participant 3]*

Additionally, when asked how they felt after participating in the cognitive ‘defusion’ exercise, Participant 18 expressed: “It doesn’t feel that different, at least for this short amount of time. I think it would take a lot longer.”

#### Adapting ACT for achievability, collaboration, and accountability

Even among those who immediately recognized the potential value of ACT, a common reaction was feeling overwhelmed about incorporating ACT into their lives due to the mental and physical loads that they manage on a regular basis:

*…I think that acceptance in general is such a huge need. I think that I would need some more help in, like, can I really apply it to everything? Like, I immediately become overwhelmed with how many different feelings I have. Like, we took it to one, but can we really do that for the hundred that go through my mind when there’s so… there’s so many? [Participant 15]*

*I feel like the thought that you can decide actions to help you live more within your values is pretty powerful. But, like you said, I feel like there’s a lot of challenges that could come up and derail those actions from happening or from you making that action happen. So it’s a lot to take in. [Participant 9]*

Participants also described ways that an ACT protocol could be tailored to better fit their needs. Regarding values and committed action, participants emphasized the importance of realistic and achievable actions, especially when considering all they are juggling as a parent to an undiagnosed child. For instance, one participant expressed:

*I think it’s hard to put into practice with the amount of time spent in and out of doctor’s appointments and the daily emails, issues, insurance, you know, it’s a difficult thing to put into practice. But could you realign and rearrange some of the goals to be not as big? Sure. Maybe you could do something, right? [Participant 11]*

Participants also voiced the importance of collaboration and accountability. They felt that having other individuals, especially other POUC, to work with would help them find value in ACT. When asked whether values and committed action may help address some of the challenges of parenting an undiagnosed child, Participant 13 responded: “I think so. Especially if you’re talking to other parents and you can bounce off ideas, that sort of thing.” Additionally, one participant underscored their need for accountability to help them follow through with practicing ACT skills:

*…I would also need some accountability where somebody is saying like, ‘Hey, did you do that?’ Because I could easily be like, ‘I totally forgot.’ And it’s so easy to go into your default of how we think versus truly being intentional with it. [Participant 15]*

In summary, participants had varied responses to ACT but ultimately expressed a resounding interest in learning more. A common reaction was feeling overwhelmed by the prospect of implementing ACT into their lives given the mental and physical demands of parenting an undiagnosed child. Nevertheless, they identified achievability, collaboration, and accountability as key elements that could support application, revealing opportunities for tailoring an ACT protocol to meet their needs.

### ACT skills group implementation: barriers, facilitators, and preferences

**Fig. 1:**
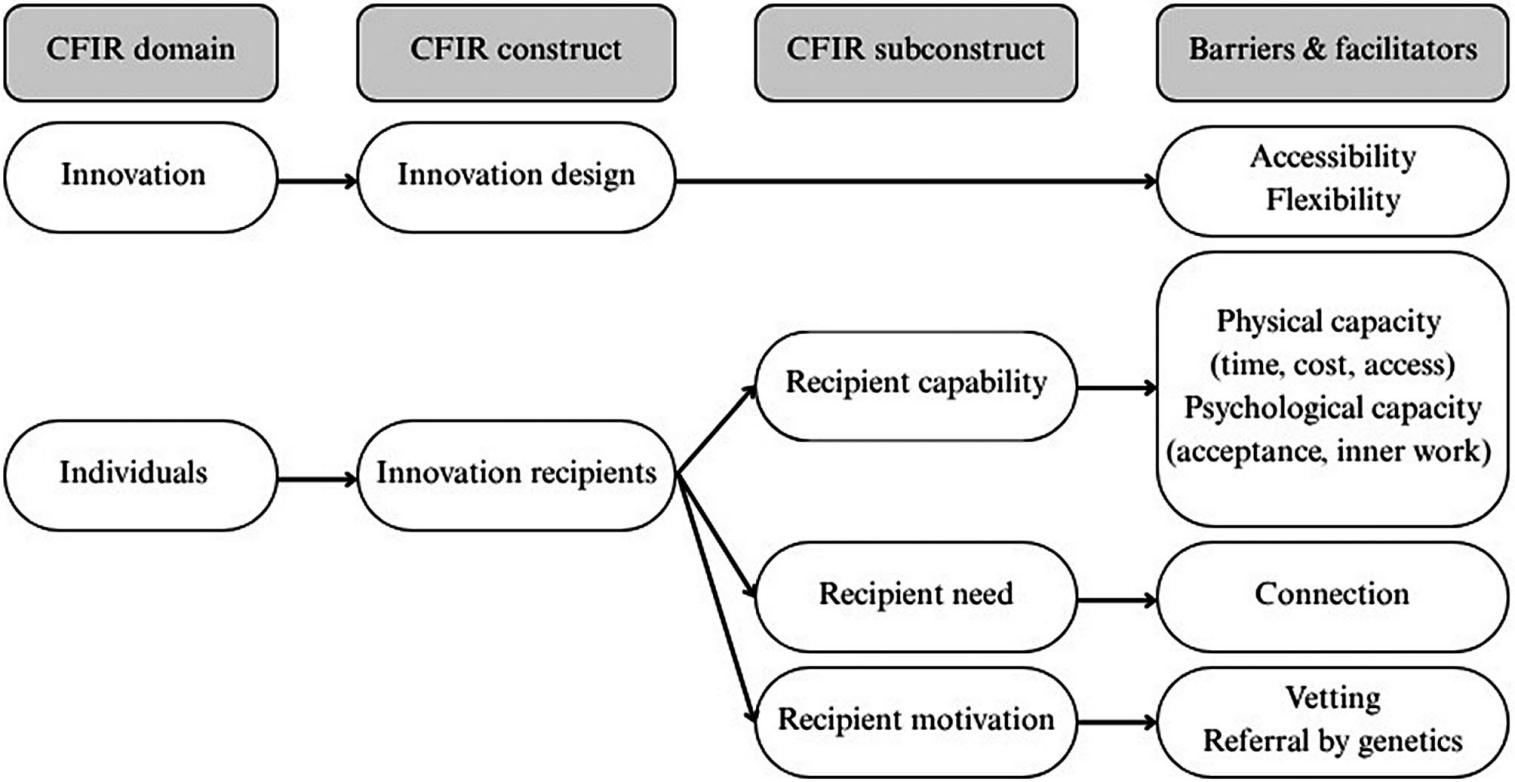
Participant-reported ACT skills group barriers and facilitators. Participant-reported barriers and facilitators to participating in an ACT skills group were mapped onto CFIR domains, constructs, and subconstructs based on which implementation determinants were most applicable

Participants discussed anticipated barriers and facilitators as well as their preferences for participating in an ACT skills group, including responses regarding setting, format, length, frequency, group size, timing, and referral mode. Their anticipated barriers and facilitators were mapped onto two main constructs from the CFIR: innovation design and innovation recipients (Fig. 1). Many of their responses were related to the design of the ACT skills group (innovation design), their physical and psychological capacity to engage in the group (recipient capability), and their specific needs and motives for participating (recipient need and recipient motivation).

#### Barriers

The most notable barriers discussed by participants were related to the CFIR recipient capability subconstruct. These barriers included time, cost, and access (Table 3).

**Table 3:**
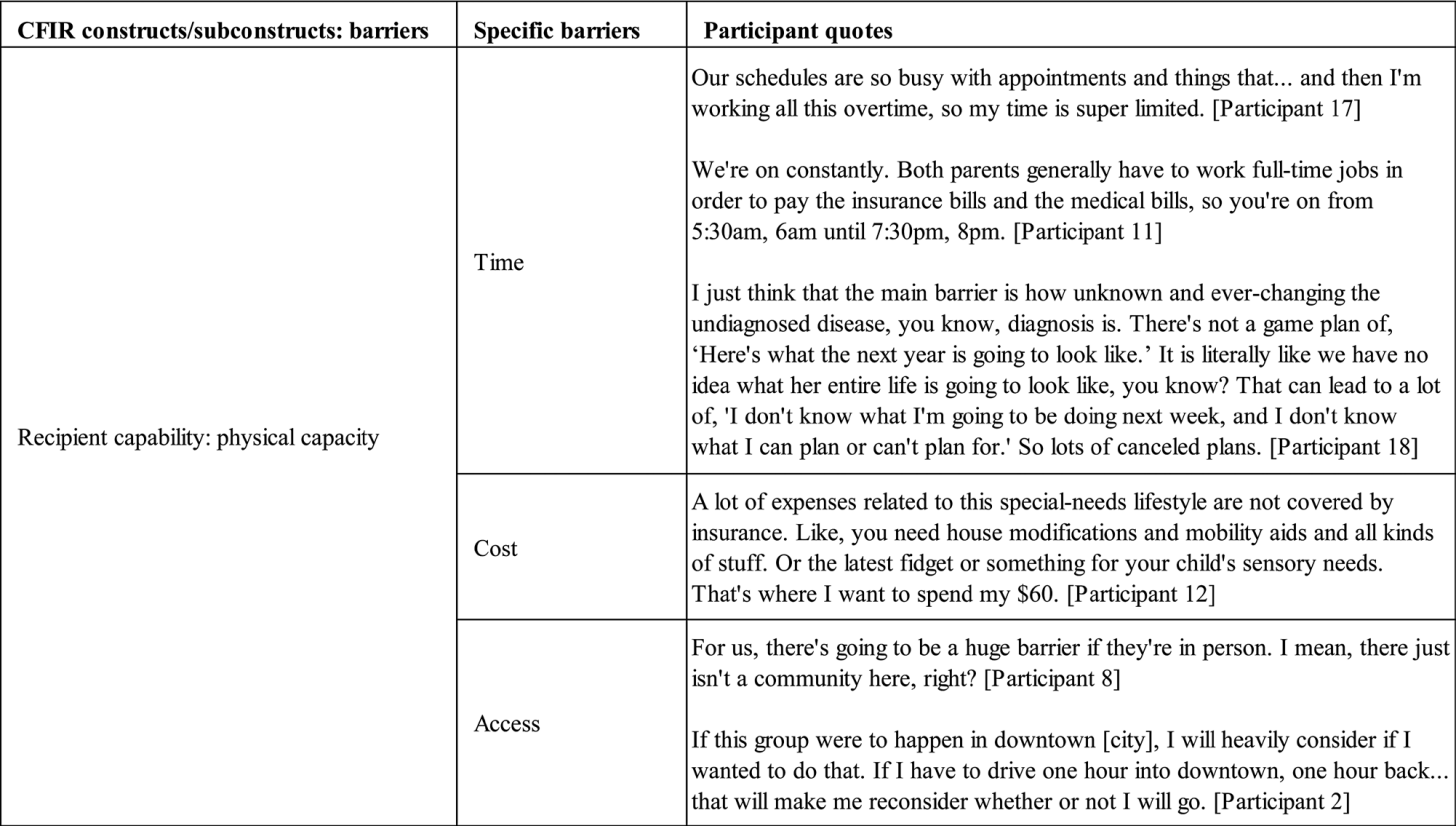
ACT skills group barriers

##### Time

Fifteen participants described lack of time as a barrier to participating in an ACT skills group. They believed their days were already full between their childcare responsibilities and their personal obligations, including work, so finding the time for an ACT skills group would be challenging.

Participants explained that they specifically struggle with the unpredictable nature of their schedules. Given that they have limited prognostic foresight for their undiagnosed child, their lives are characterized by unexpected medical appointments, hospital visits, and health crises. These unanticipated events make it difficult for them to make and keep plans outside of their undiagnosed child’s care.

Participants also discussed the challenge of finding child-free time to participate in an ACT skills group. If they were to dedicate their undivided attention to an ACT skills group, they would need to organize childcare for not only their undiagnosed child, who may have medical needs requiring specialized care, but their other children as well.

##### Cost

Seven participants described cost as a barrier to participating in an ACT skills group. They explained that the cost of accessing medical care for their undiagnosed child can be high, leaving them with limited disposable funds. Furthermore, when considering how to allocate these funds, they felt that they may be more inclined to spend on their undiagnosed child’s needs as opposed to their own.

##### Access

Finally, participants described access as a barrier to participating in an ACT skills group. Some of the access-related issues that participants described stemmed from hypothetically participating in-person versus virtually. Participants living outside of major cities explained that there is not a large community of other parents with undiagnosed children in their immediate areas, which introduces the burden of traveling long distances to an in-person group on top of the barriers previously discussed.

#### Facilitators and implementation preferences

**Table 4:**
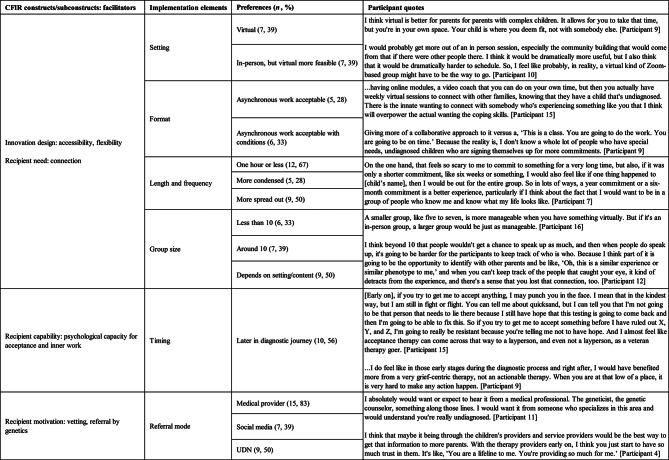
ACT skills group facilitators and implementation preferences (*n* = 18)

Participant-reported facilitators and preferences for implementing an ACT skills group are summarized in Table 4 and discussed further in this section. When describing their ideal setting, format, length, frequency, and group size, participants highlighted accessibility, flexibility, and the need for connection as facilitators. Participants also emphasized the importance of the group being easy for them to integrate into their lives, promoting forgiveness as opposed to rigidity and punitiveness, and allowing them to find support and community among other undiagnosed parents.

##### Setting

Regarding the setting of ACT skills group meetings, seven participants preferred virtual meetings over in-person meetings so they could access the group from anywhere and eliminate burdens like travel or childcare.

Another seven participants shared that although they would prefer in-person meetings for the connection aspect, virtual meetings were more feasible for them from a convenience standpoint, highlighting an important trade-off between community-building and accessibility.

##### Format

When asked if they would prefer the group format to include asynchronous work, or work that allows them to learn and practice ACT on their own time such as videos, modules, and worksheets, 11 participants responded positively to such work. However, six of the 11 participants said that certain conditions would need to be met for them to find asynchronous work acceptable, such as that a synchronous component exists to satisfy their need for connection.

Other conditions included that the asynchronous work does not require an onerous amount of time and energy, and they are not penalized if they do not complete it. Some participants cautioned against positioning the ACT skills group as a “class” because the idea that they are failing and must learn how to improve could exacerbate feelings of guilt or shame that they already struggle with as POUC.

##### Length and frequency

In terms of the length and frequency of ACT skills group meetings, 12 participants preferred one-hour meetings over meetings that lasted longer, such as two hours, as they are accustomed to this length of meetings for their undiagnosed child’s appointments and therapies. Responses were split between participants preferring ACT skills group meetings to be scheduled more condensed, such as weekly, versus less condensed, such as biweekly or monthly. Some felt that if meetings were more condensed over fewer weeks, they would be better able to plan time for them and stay consistent with their skills development. Conversely, some felt that less condensed meetings over more weeks would allow them to build stronger connections with other parents and better promote reintegration if something unexpectedly arose and they needed to miss a meeting.

Aside from the length and frequency of meetings, some participants expressed the importance of having multiple offerings for meeting dates and times so they would have a better chance of finding meetings that fit into their unpredictable schedules.

##### Group size

Responses were also split between participants preferring an ACT skills group size of less than, around, or greater than 10. However, participants generally preferred a group size of around 10 or less. Nine participants said their preferences would depend on factors like the meeting setting, whether the meetings focused more on teaching or discussion, and whether there were opportunities for more intimate conversations and relationship-building.

##### Timing

When reflecting on their preferences for the timing of the ACT skills group in the diagnostic journey, participants discussed their capacity for acceptance and inner work at different points in the journey. Ten participants shared that they would prefer to participate in a group months or years into the diagnostic journey as opposed to right away. They felt that early on, when they were in the middle of completing the genetic workup and seeking a diagnosis, they would not have been receptive to the idea of acceptance and may have even felt offended if acceptance was suggested to them before they were receptive.

Nonetheless, participants noted that they would have benefited from a resource to help them process the emotions accompanying the early stages of the diagnostic journey, such as grief, but that an actionable therapy like ACT may not have been the right fit. Additionally, they explained that later in the journey, they may have more mental capacity to dedicate themselves to self-work.

##### Referral mode

When reflecting on their preferences for being referred to or finding out about an ACT skills group, participants discussed vetting and genetics referral as important determinants for uptake. Fifteen participants shared that they would like to hear about the group from a medical provider, especially a genetics provider. They felt that genetics providers, like geneticists or genetic counselors, were most well-equipped to understand the diagnostic process and recognize the challenges of being undiagnosed.

Seven participants shared that they would like to hear about it from social media because “everybody’s on it” [Participant 17], posts are time-stamped so they know the information is current, and they can see how other parents are reacting to it. Nine participants, all of whom were recruited from UDNF PEER, shared that they would like to hear about it from sources related to the UDN. While participants discussed several different referral modes, the commonality between their responses was trust. They valued learning about the group from a trusted source that had vetted it on their behalf.

In conclusion, participants identified time constraints, financial burdens, and access challenges as the primary barriers to participating in an ACT skills group. Factors influencing their preferences for how the group is implemented included the degree to which the group is accessible and flexible, meets their need for connection, aligns with their readiness for acceptance and inner work, and is vetted by a trusted and knowledgeable source.

## Discussion

This study accomplished the objectives of (1) understanding the thoughts and emotions for which POUC most desire support, (2) assessing their prior experiences receiving mental health support, (3) exploring their reactions towards ACT, and (4) determining barriers, facilitators, and their preferences for participating in a tailored ACT skills group.

Participants described struggling with a wide array of psychosocial challenges, including ones that ACT has been shown to address by promoting psychological flexibility and values-driven living [12, 13]. Isolation emerged as the feeling for which every participant desired support. Medical practices, parent groups, advocacy organizations, and research initiatives are overwhelmingly organized around specific diagnoses. When genetic testing fails to deliver a diagnosis, POUC are left with limited access to care, community, and support—an isolating experience that even many parents of children with rare but *diagnosed* conditions do not fully understand.

Many participants reported difficulties accessing mental health practitioners that could understand their unique experiences and adequately address their needs, highlighting a critical gap in care. Incorporating psychotherapy modalities, such as ACT, into peer group formats has helped parents in similar situations [12, 13], which shows promise for parents of children with undiagnosed conditions. In fact, a systematic review by Li et al. (2023) found that group-based ACT provided more benefits than individual-based ACT for parents of children with special healthcare needs, speculating that the opportunity to exchange information and share experiences with peers introduced additional therapeutic benefits [13]. The findings from this study, especially those surrounding the importance of understanding, may help mental health practitioners to better serve POUC; however, a group-based ACT intervention for POUC could alleviate pressure on these practitioners to fully understand their unique experiences, given that peers would provide meaningful shared understanding.

Most participants recognized the potential value of ACT in the context of parenting an undiagnosed child, but many participants reported feeling overwhelmed by the prospect of incorporating ACT skills into their lives given the significant mental, emotional, and physical demands of their parenting roles. A previous study, focused on engaging individuals and families with autism spectrum disorder in mindfulness and acceptance-based therapies, similarly found that for caregivers, the length and frequency of mindfulness practices needed to be adapted around their busy lifestyles [27]. When tailoring an ACT protocol for POUC, it will be critical to think about the time and effort involved in each lesson or exercise. For example, developing mindfulness exercises that will take 30 s rather than ten minutes, or encouraging them to implement one committed action rather than several, will help equip them for success. Additionally, fostering collaboration and accountability among peers will help incentivize POUC to stay on track with their ACT skill development amidst their demanding lives.

Although ACT has been adapted to and studied in a wide variety of populations, including parents of children with special healthcare needs, chronic health conditions, etc., few studies have directly explored the barriers and facilitators to implementing ACT-based interventions for those specific populations. However, the key barriers reported in this study—time, cost and access—align with those described in literature examining barriers and facilitators to ACT and mental health service utilization in parents and caregivers of those of those with special healthcare needs [26, 27]. A scoping review by Rice et al. (2020) of the facilitators and barriers to psychosocial interventions for caregivers of people with rare diseases more broadly—not specific to parents—identified intervention characteristics (i.e. relevance, tailoring), intervention delivery (i.e. flexibility, accessibility), and support (i.e. peer involvement) as key facilitators, while key barriers included misalignment with needs, time constraints, practical barriers (i.e. travel, cost, scheduling), and emotional barriers (i.e. emotional overload) [28]. Each of these domains were discussed by participants in this study, although some barriers (i.e. needs alignment, emotional capacity) were framed as facilitators.

Given the greater logistical complexity of coordinating a group intervention compared to an individual one, it is essential to address the barriers to ACT group participation identified by POUC and tailor the intervention to their stated preferences regarding setting, format, length, frequency, group size, timing, and referral mode. For instance, due to the intense and often unpredictable nature of their schedules, an ACT skills group must be delivered in a time-efficient manner. This may involve developing asynchronous instructional materials that allow POUC to learn ACT skills at their own convenience, thereby preserving synchronous meeting time for interactive, connection-building activities such as group practice, discussion, and reflection. Although no asynchronous ACT-based interventions have been specifically studied in POUC or related parent populations, existing research demonstrates that ACT can be effective when delivered asynchronously (i.e. bibliotherapy) and with minimal therapeutic support [29, 30]. Additionally, given the financial constraints many POUC face due to substantial medical expenses, affordability must be maximized. One cost-reduction strategy may involve utilizing free, widely accessible platforms, such as Zoom, for synchronous sessions, thereby avoiding expenses associated with proprietary software. Another approach may be recruiting individuals to facilitate the group on a volunteer basis. Eliminating facilitator compensation may lower the financial burden on participants by reducing overall implementation costs.

Many participants suggested that offering an ACT skills group virtually would help with accessibility; however, they weighed this preference against their desire for connection, expressing that an in-person group would feel more connected. In a study by Lopez et al. (2020) comparing group cohesion between patients who participated in a dialectical behavior therapy group via video teleconferencing and an in-person group, the patients in the video group reported significantly lower levels of connection with fellow group members compared to those in the in-person group. However, the video group demonstrated a higher average attendance rate compared to the in-person group. Importantly, the participants in Lopez et al. (2020) expressed that the convenience of the video group outweighed any negative benefits of not meeting in person, which aligns with participants in this study [31]. Although virtual groups may be less effective in facilitating relationship building, they offer increased accessibility. Therefore, it is crucial to explore strategies that may help foster connection in a virtual ACT skills group. Strategies may include leveraging technological features such as breakout rooms, chat functions, screen sharing, or polls as well as implementing structured opportunities for personal sharing and small-group interaction [31, 32].

Interestingly, during participant discussions regarding acceptance and the ideal timing of participating in an ACT skills group, some interpreted the concept of acceptance as a directive to stop seeking answers or advocating for their undiagnosed child. This interpretation may have stemmed from implicit associations with the term *acceptance* within the context of their child’s diagnostic journey, or from only receiving a brief introduction to the concept of acceptance during their interviews. Rather, ACT encourages accepting, as opposed to fighting, difficult thoughts and emotions to diminish their power; a practice that can be utilized by POUC regardless of their stage in the diagnostic journey and beyond, not only by those who are ready to accept their child’s undiagnosed status. When considering how to disseminate information about the group, it will be important to ensure the goals of ACT, especially those related to acceptance, are conveyed thoughtfully. For example, the term “acceptance” may be replaced with “willingness.” Although both terms have been used to describe the same psychological process within the ACT framework, “willingness” may accurately convey the intended meaning without evoking potential misinterpretations [33]. Additionally, some participants explained that they may not have had the capacity to focus on self-work when they were at the beginning of the diagnostic journey due to focusing so much energy on their undiagnosed child and coping with strong emotions such as grief, loss, and uncertainty. Nonetheless, they felt that support was needed during that time. Thus, for POUC in earlier stages, a psychotherapy approach that is more support-based versus action-based, such as client-centered therapy or emotion-focused therapy, may be more appropriate [34, 35]. This consideration must be at the forefront when developing strategies for identifying appropriate parents to participate in an ACT skills group.

In summary, the findings from this study will inform the development of a tailored ACT protocol and guide the implementation of an ACT-based skills group designed specifically for POUC. In addition, this study contributes to the literature more broadly by identifying key barriers and facilitators that influence access to resources for this population, both within the domain of mental health and across other dimensions of care. Importantly, this research will help to equip healthcare providers with concrete, evidence-based mental health resources to offer POUC, particularly at critical junctures when diagnostic roadblocks create distress.

### Study strengths and limitations

A key strength of this study is that it was grounded in implementation science, leveraging the CFIR to develop interview questions, guide data analysis, and organize barriers and facilitators into constructs. As a result, it is clear which CFIR constructs were explored in this study, and which require further studying. Another strength is that participants were exposed to ACT, which allowed the study team to gauge their initial reactions to ACT and gain more informed insights for implementing an ACT skills group. However, the development of psychological flexibility via ACT is a gradual process that involves sustained practice and deep self-reflection [10, 11]. As such, this pre-implementation study cannot fully capture how participants may respond to ACT over time or the potential benefits they may derive from completing an ACT skills group. Additionally, recruitment for this study took place across multiple avenues, including a single hospital system, social media, and a nationwide peer support group. This multifaceted recruitment strategy facilitated the inclusion of POUC from across the United States with diverse experiences, enhancing the richness of the sample. Despite diversity from this perspective, participants predominantly identified as mothers, women, married, white, and working. The results of this study may not adequately capture the perspectives of individuals who identify differently, such as fathers or non-parent caregivers as well as mothers who are non-white, single, etc. Furthermore, POUC with limited access to healthcare and research programs may not be appropriately represented in the study, and may desire support for different psychosocial experiences or face more significant barriers to participating in an ACT skills group Finally, the majority of participants reported prior experience receiving mental health therapy, which could have skewed their views on ACT and mental health therapy in general compared with the general population of POUC.

### Research recommendations

Although this study captured the perspectives of POUC, gathering input from other key constituents, such as institutional or organizational leaders and potential group facilitators, is essential to support the successful implementation of an ACT skills group for this population. Participants discussed the importance of genetics providers, such as genetic counselors, being involved in ACT skills group dissemination efforts. To go a step further, genetic counselors may be particularly well-suited to serve as group facilitators due to their training in effective and empathetic communication, expertise in genetics, placement within diagnostic research programs, and familiarity with the diagnostic journey that POUC often navigate. Despite their many qualifications, genetic counselors, and any non-psychotherapy providers, would require special training to learn how to facilitate an ACT skills group. Studies have demonstrated the feasibility of training non-psychotherapy providers to deliver ACT, including a study by Collision K, Lawrence E, Ofodu C, Hasan T, and Goldstein Z (2025) in which probation officers were trained to administer an ACT intervention to individuals on probation or parole [unpublished]. As such, future research may explore genetic counselor perspectives and insights regarding their potential roles within this intervention. This study focused specifically on barriers and facilitators within the intervention design and intervention recipient constructs of the CFIR framework, but future research may examine additional CFIR domains and constructs to develop a more comprehensive understanding of implementation challenges and opportunities. Moreover, there is a critical need to capture the experiences of a more diverse sample of parents, particularly those with limited access to healthcare and research programs, to ensure the intervention is equitable to all who may benefit from it. Implementation of an ACT skills group for this population must follow an iterative process. As this is a pre-implementation study, subsequent research may focus on pilot implementation, including feasibility trials and experimenting with different implementation variables. Mixed-method approaches may be utilized to evaluate mental health outcomes following participation and continue adapting the intervention based on participant feedback. Future work may also assess whether the initial responses to ACT observed in this study align with longer-term perspectives and further investigate whether ACT is an appropriate therapeutic approach for this population.

## Conclusions

This pre-implementation study described the psychosocial challenges for which POUC most desire support and the potential for ACT to improve these issues by increasing psychological flexibility and values-based living. Participants highlighted their experiences of isolation and difficulties in accessing psychotherapy that truly supports their unique needs. Although participants expressed varied reactions to ACT, there was overall interest in exploring it further, provided it is adapted to meet their specific needs. Key considerations for implementing an ACT skills group for this population included enhancing accessibility and flexibility, fostering connection, and involving trusted sources and genetics providers in dissemination efforts while also minimizing barriers related to time, cost, and access. Future research and pilot implementation studies are needed to evaluate outcomes and continue refining a tailored ACT skills group protocol and implementation strategy that is effective and accessible for all POUC.

## Supplementary Information

Below is the link to the electronic supplementary material.

Supplementary Material 1

## Data Availability

The datasets generated and/or analyzed during the current study are not publicly available due to privacy protections but are available from the corresponding author on reasonable request.

## Declarations

### Author information

This study was completed in fulfillment of SR's thesis requirement for the Graduate Program in Genetic Counseling at Northwestern University.

## References

[1] Sullivan JA, Schoch K, Spillman RC, Shashi V. Exome/Genome Sequencing in Undiagnosed Syndromes. Annu Rev Med. 2023;74:489–502. 10.1146/annurev-med-042921-110721. 36706750 10.1146/annurev-med-042921-110721 PMC10483513

[2] Kohler J, Turbitt E, Biesecker B. Personal utility in genomic testing: a systematic literature review. Eur J Hum Genet. 2017;25(6):662–8. 10.1038/ejhg.2017.10. 28295040 10.1038/ejhg.2017.10 PMC5477355

[3] McConkie-Rosell A, Hooper SR, Pena LDM, et al. Psychosocial Profiles of Parents of Children with Undiagnosed Diseases: Managing Well or Just Managing? J Genet Couns. 2018;27(4):935–46. 10.1007/s10897-017-0193-5. 29297108 10.1007/s10897-017-0193-5 PMC6028305

[4] Aldiss S, Gibson F, Geoghegan S, et al. We don’t know what tomorrow will bring’: Parents’ experiences of caring for a child with an undiagnosed genetic condition. Child Care Health Dev. 2021;47(5):588–96. 10.1111/cch.12866. 33709393 10.1111/cch.12866

[5] Atkins JC, Padgett CR. Living with a Rare Disease: Psychosocial Impacts for Parents and Family Members – a Systematic Review. J Child Fam stud. 2024;33:617–36. 10.1007/s10826-024-02790-6.

[6] Bauskis A, Strange C, Molster C, Fisher C. The diagnostic odyssey: insights from parents of children living with an undiagnosed condition. Orphanet J Rare Dis. 2022;17(1):233. 10.1186/s13023-022-02358-x. 35717227 10.1186/s13023-022-02358-x PMC9206122

[7] Gurasashvili J, Silverio SA, Hill M, Peter M, Stafford-Smith B, Lewis C. The disequilibrium of hope: A grounded theory analysis of parents’ experiences of receiving a no primary finding result from genome sequencing. J Genet Couns. 2024;33:1089–102. 10.1002/jgc4.1818. 37929616 10.1002/jgc4.1818

[8] Lewis C, Skirton H, Jones R. Living without a diagnosis: the parental experience. Genetic Test Mol Biomarkers. 2010;14(6):807–15. 10.1089/gtmb.2010.0061. 10.1089/gtmb.2010.0061 20939735

[9] Spillmann R, McConkie-Rosell A, Pena L, et al. A window into living with an undiagnosed disease: illness narratives from the Undiagnosed Diseases Network. Orphanet J Rare Dis. 2017;12(1):71. 10.1186/s13023-017-0623-3. 28416019 10.1186/s13023-017-0623-3 PMC5392939

[10] Hayes SC, Luoma JB, Bond FW, Masuda A, Lillis J. Acceptance and commitment therapy: model, processes and outcomes. Behav Res Ther. 2006;44(1):1–25. 10.1016/j.brat.2005.06.006. 16300724 10.1016/j.brat.2005.06.006

[11] Hayes SC, Barnes-Holmes D, Wilson KG. Acceptance and commitment therapy and contextual behavioral science: examining the progress of a distinctive model of behavioral and cognitive therapy. Behav Res Ther. 2013;44(2):180–98. 10.1016/j.beth.2009.08.002. 10.1016/j.beth.2009.08.002 PMC3635495 23611068

[12] Byrne G, Ní Ghráda Á, O’Mahony T, Brennan E. A systematic review of the use of acceptance and commitment therapy in supporting parents. Psychol Psychotherapy: Theory Res Pract. 2021;94(Suppl 2):378–407. 10.1111/papt.12282. 10.1111/papt.12282 32406169

[13] Li S, Chen Z, Yong Y, Xie J, Li Y. Effectiveness of acceptance and commitment therapy-based interventions for improving the psychological health of parents of children with special health care needs: A systematic review and meta-analysis. Compr Psychiatr. 2023;127:152426. 10.1016/j.comppsych.2023.152426. 10.1016/j.comppsych.2023.152426 37757593

[14] Danino M, Shechtman Z. Superiority of group counseling to individual coaching for parents of children with learning disabilities. Psychother Research: J Soc Psychother Res. 2012;22(5):592–603. 10.1080/10503307.2012.692953. 10.1080/10503307.2012.692953 22694319

[15] Chakraborti M, Gitimoghaddam M, McKellin WH, Miller AR, Collet J–P. Understanding the implications of peer support for families of children with neurodevelopmental and intellectual disabilities: a scoping review. Front Public Health. 2021;9:719640. 10.3389/fpubh.2021.719640. 34888278 10.3389/fpubh.2021.719640 PMC8649771

[16] Lancaster K, Kern ML, Harding K, et al. Exploring long-term outcomes of a peer support programme for parents of children with disability in Australia. Child Care Health Dev. 2024;50(2):e13236. 10.1111/cch.13236. 38426583 10.1111/cch.13236

[17] Nilsen P. Making sense of implementation theories, models and frameworks. Implement Sci. 2015;10:53. 10.1186/s13012-015-0242-0. 25895742 10.1186/s13012-015-0242-0 PMC4406164

[18] Damschroder LJ, Reardon CM, Widerquist MAO, Lowery J. The updated Consolidated Framework for Implementation Research based on user feedback. Implement Sci. 2022;17:75. 10.1186/s13012-022-01245-0. 36309746 10.1186/s13012-022-01245-0 PMC9617234

[19] Tong A, Sainsbury P, Craig J. Consolidated criteria for reporting qualitative research (COREQ): a 32-item checklist for interviews and focus groups. Int J Qual Health Care. 2007;19(6):349–57. 10.1093/intqhc/mzm042. 17872937 10.1093/intqhc/mzm042

[20] Silverman D. Interpreting qualitative data: methods for analyzing talk, text, and interaction. 2nd edition. Thousand Oaks (CA): Sage Publications; 2001.

[21] Association for Contextual Behavioral Science. Treatment protocol and manuals [Internet]. Available from: https://contextualscience.org/treatment_protocol_and_manuals

[22] Dedoose, Version. 9.2.14, cloud application for managing, analyzing, and presenting qualitative and mixed method research data. Los Angeles, CA: SocioCultural Research Consultants, LLC www.dedoose.com; 2025.

[23] MacQueen KM, McLellan E, Kay K, Milstein B. Codebook development for team-based qualitative analysis. Field Methods. 1998;10(2):31–6. 10.1177/1525822X9801000203.

[24] Cicchetti DV. Guidelines, criteria, and rules of thumb for evaluating normed and standardized assessment instruments in psychology. Psychol Assess. 1994;6(4):284–90. 10.1037/1040-3590.6.4.284.

[25] Terry G, Hayfield N, Clarke V, Braun V. Thematic analysis. In: Willig C, Stainton Rogers W, editors. The SAGE handbook of qualitative research in psychology. 2nd ed. London: SAGE Publications Ltd; 2017. pp. 17–37. https://methods.sagepub.com/book/the-sage-handbook-of-qualitative-research-in-psychology-second-edition.

[26] Graaf G, Baiden P, Keyes L, Boyd G. Barriers to Mental Health Services for Parents and Siblings of Children with Special Health Care Needs. J Child Fam stud. 2022;31:881–95. 10.1007/s10826-022-02228-x. 35039741 10.1007/s10826-022-02228-x PMC8754365

[27] Hartley M, Due C, Dorstyn D. Barriers and facilitators to engaging individuals and families with autism spectrum disorder in mindfulness and acceptance-based therapies: a meta-synthesis. Disabil Rehabil. 2021;44(17):4590–601. 10.1080/09638288.2021.1921859. 34033733 10.1080/09638288.2021.1921859

[28] Rice DB, Carboni-Jiménez A, Cañedo-Ayala M, et al. Perceived benefits and facilitators and barriers to providing psychosocial interventions for informal caregivers of people with rare diseases: a scoping review. Patient. 2020;13:471–519. 10.1007/s40271-020-00441-8. 32785886 10.1007/s40271-020-00441-8

[29] Keenan E, Muldoon OT, Boylan C, Curry P, McCaul K. A qualitative feasibility and acceptability study of an acceptance and commitment-based bibliotherapy intervention for people with cancer. J Health Psychol. 2024;29(5):410–24. 10.1177/13591053231216017. 38158736 10.1177/13591053231216017 PMC11005316

[30] Veillette J, Martel M-E, Dionne F. A randomized controlled trial evaluating the effectiveness of an acceptance and commitment therapy-based bibliotherapy intervention among adults living with chronic pain. Can J Pain. 2019;3(1):209–25. 10.1080/24740527.2019.1678113. 35005411 10.1080/24740527.2019.1678113 PMC8730660

[31] Lopez A, Rothberg B, Reaser E, Schwenk S, Griffin R. Therapeutic groups via video teleconferencing and the impact on group cohesion. mHealth. 2020;6:13. 10.21037/mhealth.2019.11.04. 32270005 10.21037/mhealth.2019.11.04 PMC7136655

[32] Havlik S, Malott KM, Gamerman T, Okonya P. Working across differences while online: examining the experience of facilitating a virtual group. Int J Advancement Couns. 2023;45:291–309. 10.1007/s10447-022-09496-8. 10.1007/s10447-022-09496-8 PMC9685035 36466590

[33] Hayes SC. Acceptance and commitment therapy, relational frame theory, and the third wave of behavioral and cognitive therapies – republished article. Behav Res Ther. 2016;47(6):869–85. 10.1016/j.beth.2016.11.006. 10.1016/j.beth.2016.11.006 27993338

[34] Kirschenbaum H, Jourdan A. The current status of Carl Rogers and the person-centered approach. Psychother Theory Res Pract Train. 2005;42(1):37–51. 10.1037/0033-3204.42.1.37.

[35] Marren C, Mikoška P, O’Brien S, Timulak L. A qualitative meta-analysis of the clients’ experiences of emotion-focused therapy. Clin Psychol Psychother. 2022;29(5):1611–25. 10.1002/cpp.2745. 35491475 10.1002/cpp.2745

