# Supplementary material for: Tailoring acceptance and commitment therapy for parents of children with undiagnosed conditions: a qualitative pre-implementation study
Source: Orphanet J Rare Dis. 2026 May 2;21:234. doi: 10.1186/s13023-026-04355-w (PMC13317163; doi:10.1186/s13023-026-04355-w)
Supplement: Supplementary file 1 — Supplementary Material 1 [file 13023_2026_4355_MOESM1_ESM.docx]

**Tailoring acceptance and commitment therapy for parents of children with undiagnosed conditions: A qualitative pre-implementation study**

Interview Guide

| Aim: Understand the thoughts and emotions that parents of children with undiagnosed conditions most desire support for and their prior experience with mental health support. |
| --- |

Being a parent comes with a range of both positive and negative emotions, and being a parent to a child with an undiagnosed condition is no exception. Parenting a child with an undiagnosed condition comes with unique challenges, which can evoke feelings of stress, anxiety, overwhelm, uncertainty, lack of control, loss of self, hopelessness, isolation, anger, frustration, among many others. I would like to start by talking about your perspectives on mental health support in helping you manage difficult emotions like these.

Main questions:

- Reflecting on your experience as a parent of a child (or children) with an undiagnosed condition, what thoughts or emotions would you most like a mental health support resource to address?
- Have you ever used mental health support resources to address challenges related to being a parent of a child (or children) with an undiagnosed condition?
  - What kind of resources have you used?
  - If so, what was that like? In what ways was that helpful or unhelpful?
  - If not, why not? Do you anticipate that would be helpful or unhelpful?

| Aim: Explore the perspectives of parents of children with undiagnosed conditions towards ACT concepts and exercises. |
| --- |

Acceptance:

To illustrate the first ACT concept, I would like to lead you through an exercise:

Are you familiar with quicksand? *(ensure they are familiar)* While you likely have never encountered quicksand in real life, you have probably seen it on television or in movies. Now, imagine what you would do if you got caught in quicksand. What would your basic instincts tell you to do? (elicit responses like jumping, flailing, etc.) While those are natural responses to wanting to get out of quicksand, that is the last thing that you should do. When you try to struggle your way out of quicksand, you sink even deeper into it. Have you ever heard what you are recommended to do if you get caught in quicksand? *(elicit responses like relax, stay calm, etc.)* Exactly, you need to relax, stop struggling, and lie back into the quicksand so that more of your body is in contact with it. If you do that, you will float to the top where you can stay until help arrives.

Quicksand is a great example of how sometimes, continuing to struggle is doing more harm than good. That brings us to an important concept in ACT, which is acceptance. Acceptance is the willingness to experience, rather than fight, difficult emotions. The desire to fight is natural, but when it comes to difficult emotions, fighting them only causes you to pay more attention and devote more energy to them.

There are some different skills in ACT that can help you accept, rather than fight, difficult emotions. I will lead you through an exercise to illustrate one of these skills:

You can turn off your camera for this exercise if it makes you feel more comfortable. Please feel free to close your eyes if you’d like. Now, please think of a particularly sticky and hard to release thought that you have – maybe it is a judgement you make on yourself as a parent or as a person. Take a moment now to think of it. First, let the thought set into you. Repeat the thought in your mind and try to really buy into it.

Now, I will ask to you repeat the thought in your mind but start with “I am having the thought that….” For instance, if my thought is, “I am not doing enough,” I would instead think, “I am having the thought that I am not doing enough.” Please practice this for a few moments.

Thank you, you can open your eyes and turn your camera back on. This exercise is an example of cognitive defusion, or creating distance between yourself and your thoughts. It is the idea that thoughts are just thoughts, and they can only control you if you allow them to. Acceptance, and skills like cognitive defusion, are important ACT concepts that would be discussed in much more depth in an ACT skills group.

Main questions:

- What thoughts did you have while going through the quicksand and cognitive defusion exercises?
- What thoughts do you have after going through those exercises?
- What do you think about acceptance in helping address the thoughts and emotions that come with parenting a child (or children) with an undiagnosed condition?
- Would learning more skills for accepting difficult thoughts and emotions, such as cognitive defusion, be useful? Why or why not?

Values and Committed Action:

To illustrate a couple more ACT concepts, I would like to lead you through an exercise:

I am placing a list of values on the screen – this is not a comprehensive list but is meant to give you some examples. Whether we are cognizant of them or not, we all have values. Some characteristics of values are that they are what you want to live for and embody, they are personal and important to you, they are chosen freely, and they give direction and meaning to life. It is worth noting that values are different from goals in that they are never fully achieved. Please take a moment to look over this list and choose one value that is important to you and that you want to work on. I will give you a few moments – please let me know once you've chosen.

Now, think about actions you can take to live in alignment with that value. Try to think of at least one short-term action, or an action you could complete today or this week, and one long-term action, or an action you could work on over the course of months or years. For instance, if the value I chose is service, for the short-term action, I may commit to volunteering with an organization that is meaningful to me once a week. For the long-term action, I may commit to applying for a leadership role within the organization within the next year.

This exercise can be done for other values and can be specific to certain areas of your life, such as family, career, health, etc. Now, what if difficult thoughts or emotions tried getting in the way of you carrying out these actions? That is where committed action comes into play. Committed action refers to acting in alignment with your values, regardless of difficult thoughts or emotions. Values and committed action are two important ACT concepts that would be discussed in much more depth in an ACT skills group.

Main questions:

- What thoughts did you have while going through the values exercise?
- What thoughts do you have after going through the exercise?
- What do you think about committed action to help address the difficult thoughts and emotions that come with parenting a child (or children) with an undiagnosed condition?
- Would learning skills for practicing committed action be useful? Why or why not?

Aim: Determine barriers, facilitators, and preferences for participating in an ACT skills group for parents of children with undiagnosed conditions.

To start the last topic of conversation, I understand that you likely have a lot on your plate, especially as a parent caring for a child with complex needs. As such, I would like to hear your perspective on how an ACT skills group could be implemented in a way that makes sense for you and fits into your life. After briefly reviewing some ACT concepts and going through a few exercises, you may be uncertain about whether you would participate in an ACT skills group, which is completely fine! I invite you to answer these questions as if you were interested in participating.

Main question:

- What barriers do you think would prevent you from participating in an ACT skills group?

Thank you for sharing. Now, let’s discuss how these barriers could be overcome:

Main question:

- What would make it easiest for you to participate in an ACT skills group? You may comment on the number of meetings, length of meetings, setting of meetings (virtual, in person, etc.), number of participants in the group, or anything else that comes to mind.

Probing Questions:

- When would it be preferable for you to participate in an ACT skills group? While going through the genetic testing process, or months/years after?
- What do you think about participating in three, two-hour meetings that span over three weeks? Would you prefer longer or shorter meetings? More condensed or more spread out meetings?
- Would you rather participate in an ACT skills group in-person or virtually?
- What group size would be preferable? Groups of 5, 10, 20, a different number?
- What would be the best way for you to learn about or receive a referral for an ACT skills group? Through a medical professional, rare/undiagnosed disease organization, social media group, etc.?

Values List

Accountability - Achievement - Adaptability - Adventure - Altruism Ambition - Authenticity - Balance - Beauty - Being the best - Belonging Career - Caring - Collaboration - Commitment - Community Compassion - Competence - Confidence - Connection - Contentment Contribution - Cooperation - Courage - Creativity - Curiosity - Dignity Diversity - Environment - Efficiency - Equality - Ethics - Excellence Fairness - Faith - Family - Financial stability - Forgiveness - Freedom Friendship - Fun - Future generations - Generosity - Giving back - Grace Gratitude - Growth - Harmony - Health - Home - Honesty - Hope Humility - Humor - Inclusion - Independence - Initiative - Integrity Intuition - Job security - Joy - Justice - Kindness - Knowledge Leadership - Learning - Legacy - Leisure - Love - Loyalty - Making a difference - Nature - Openness - Optimism - Order - Parenting - Patience Patriotism - Peace - Perseverance - Personal fulfillment - Power - Pride Recognition - Reliability - Resourcefulness - Respect - Responsibility Risk-taking - Safety - Security - Self-discipline - Self-expression - Self-respect - Serenity - Service - Simplicity - Spirituality - Sportsmanship Stewardship - Success - Teamwork - Thrift - Time - Tradition - Travel Trust - Truth - Understanding - Uniqueness - Usefulness - Vision Vulnerability - Wealth - Well-being - Wholeheartedness - Wisdom
